# Inpatient Nurses’ Anticipatory Perspectives on Assistive Robots: Qualitative Study

**DOI:** 10.2196/91106

**Published:** 2026-08-10

**Authors:** Mavis Weiting Tan, Timie Lai Teng Chan, Charmaine Shi Min Taye

**Affiliations:** 1 Department of Nursing, Woodlands Hospital, 17 Woodlands Drive,Singapore, 737628, Singapore, 65 6363 3000

**Keywords:** nurses, assistive robots, human-robot interaction, preimplementation, technology adoption

## Abstract

**Background:**

Assistive robots have been proposed to support nursing work by offloading routine, logistical, and physically demanding tasks. However, existing research has largely focused on postimplementation acceptance or usability, with fewer studies examining how nurses conceptualize assistive robots before routine deployment, particularly in settings where exposure remains limited.

**Objective:**

This study aimed to explore inpatient nurses’ anticipatory perspectives on assistive robots and to examine how perceived benefits, concerns, and implementation conditions are constructed at the preimplementation stage.

**Methods:**

A qualitative descriptive study was conducted using semistructured, face-to-face interviews with 16 registered nurses aged 21 to 32 years from 4 general medical wards in a public tertiary hospital in Singapore. At the time of data collection, assistive robots had not been routinely implemented; participants’ exposure was limited to pilots, demonstrations, and indirect sources. Interview transcripts were analyzed using reflexive thematic analysis.

**Results:**

Participants’ accounts reflected anticipatory acceptance characterized by cautious optimism regarding the potential of assistive robots to support nursing work, alongside concurrent concerns about safety, professional identity, relational care, and implementation feasibility. Four themes were developed: anticipatory optimism shaped by indirect exposure; negotiating uncertainty, safety, and professional boundaries; imagining assistive robots as workload and workflow support; and conditions for responsible implementation. Concerns centered on professional identity, patient safety, ethical boundaries, loss of human touch, technical reliability, cost-effectiveness, and organizational feasibility. Participants viewed assistive robots as acceptable only if they were integrated into nursing workflows, preserved clinical judgment and relational care, and were supported by training, technical infrastructure, leadership commitment, and clear governance.

**Conclusions:**

Nurses’ anticipatory perspectives suggest that acceptance of assistive robots depends not only on perceived usefulness, but also on alignment with professional values, patient safety, relational care, and organizational readiness. Early nurse involvement, human-centered implementation, structured training, and clear governance are needed to ensure that assistive robots augment rather than undermine nursing practice.

## Introduction

### Assistive Robots and Their Emergence in Health Care

Assistive robots are increasingly positioned within the broader category of service assistive robots. Service assistive robots are commonly defined as assistive robots for personal or professional use that perform useful tasks for humans or equipment, excluding industrial automation applications [1]. In health care, assistive robots have been applied in multiple areas, including rehabilitation, patient mobility support, logistics, medication or supply delivery, socially assistive companionship, monitoring, and patient-facing support functions [2-6].

Interest in assistive robots has grown in response to workforce shortages, aging populations, infection-control demands, and pressure to improve productivity in health care systems [7-9]. Developments in sensor technology, AI, autonomous navigation, and human-robot interaction have expanded the potential functions of assistive robots beyond fixed repetitive tasks toward more adaptive forms of clinical and social support [2-4]. These capabilities not only make assistive robots potentially useful in care environments but also raise questions about safety, accountability, workflow integration, and the boundaries of human-centered care [4,10,11].

During the COVID-19 pandemic, socially assistive robots were deployed in some care settings to support symptom screening and exposure assessment, illustrating one way assistive robots were used to reduce infection-related contact while maintaining service delivery [12,13]. However, pandemic-related deployments also highlighted that assistive robots must be evaluated not only for technical feasibility but also for acceptability, workflow fit, and their effects on human interaction [12,13].

Within nursing contexts, the term “assistive robots” refers to robots designed to support care through routine, physical, logistical, automated, or interactive functions. These include assistive robots and automated devices used in nurses’ work, physically assistive technologies, and socially assistive robots intended to support interaction, engagement, or care delivery [4-6,10]. By reducing physical strain and repetitive workloads, assistive robots may help support nursing work and preserve time for patient-centered care [4,5,10]. However, their introduction into nursing practice is not merely a technical intervention. It may reshape task ownership, professional responsibility, nurse-patient interaction, and perceptions of what constitutes appropriate care [4,10,11].

### Technology Acceptance and Preimplementation Perspectives

Technology acceptance research provides a useful starting point for understanding how health professionals respond to emerging technologies. The Technology Acceptance Model (TAM) and its extensions, including the Unified Theory of Acceptance and Use of Technology (UTAUT), emphasize constructs such as perceived usefulness, perceived ease of use, social influence, facilitating conditions, and intention to use [14,15]. In robot acceptance research, the Almere Model further adapts technology acceptance concepts for assistive and socially assistive robots by incorporating constructs such as trust, anxiety, social presence, perceived enjoyment, and perceived sociability [16].

However, acceptance is not formed solely through direct use. In preimplementation contexts, health professionals may construct expectations through demonstrations, workplace discourse, media narratives, peer accounts, or limited observational exposure. These anticipatory judgments can influence whether a technology is perceived as useful, safe, legitimate, or compatible with professional values before it becomes part of routine practice [15-18].

In this study, these anticipatory judgments are conceptualized as *anticipatory acceptance*, referring to nurses’ prospective evaluation of whether assistive robots are likely to be useful, safe, appropriate, and compatible with nursing practice before routine implementation occurs. Unlike postimplementation technology acceptance, which develops through direct interaction and accumulated user experience, anticipatory acceptance is informed by indirect exposure and expectations about future clinical practice. It is also distinct from organizational readiness, which reflects preparedness for implementation, and from general attitudes toward technology, which do not necessarily consider how an innovation may be integrated into everyday nursing work. By focusing on anticipatory acceptance, this study seeks to understand how nurses begin to negotiate the potential role of assistive robots in nursing practice before assistive robots become part of routine care.

In nursing, the preimplementation stage is especially important because assistive robots enter a practice environment where clinical judgment, embodied care, emotional labor, accountability, and workflow coordination are central [4,10,11]. Nurses’ early interpretations may therefore shape not only their willingness to use assistive robots but also how they define appropriate task delegation, human oversight, and acceptable limits of automation.

### Nurses’ Acceptance of Assistive Robots

Existing studies suggest that nurses and care professionals often hold cautiously positive attitudes toward assistive robots [6,13,17-20]. Positive perceptions are commonly associated with the potential to reduce physical strain, support routine or repetitive tasks, improve workflow efficiency, and alleviate aspects of the nursing workload [4-6,10]. At the same time, concerns persist regarding technical reliability, patient safety, the loss of human touch, privacy, ethical accountability, costs, and potential effects on employment or professional identity [6,10,11,13,17-20].

Acceptance also appears to be shaped by task type and perceived fit with professional values. Assistive robots tend to be viewed as more acceptable for logistical, repetitive, or physically demanding tasks than for emotionally sensitive, relational, or judgment-intensive aspects of care [5,6,13,17,20]. This distinction is important in nursing because care work is not only defined by task completion but also by surveillance, interpretation, communication, and relational presence [10,11].

The work by Turja et al [17,18] is particularly relevant because it moves beyond general technology acceptance to examine care workers’ readiness for robotization and their intention to use assistive robots in professional contexts. Their Robot Acceptance Model for Care (RAM-care) highlights that robot acceptance is shaped not only by perceived usefulness but also by social influence, attitudes, and whether robot use is perceived as consistent with personal and professional values [17]. This suggests that acceptance of assistive robots in nursing cannot be understood as an individual preference alone; it is also shaped by organizational expectations, task legitimacy, perceived value alignment, and the wider social meaning of robotized care [17,18].

Despite this growing literature, important gaps remain. Much of the existing evidence is quantitative, cross-sectional, or based on attitude and acceptance measures [17-20]. These studies are useful for identifying predictors of acceptance, but they provide limited insight into how nurses interpret, negotiate, and anticipate the implications of assistive robots before routine implementation. Existing qualitative work has begun to examine nurses’ experiences and perceptions of socially assistive technologies, including during COVID-19-related deployments [13], but fewer studies have explored anticipatory perspectives in acute inpatient settings where assistive robots remain emergent rather than embedded. This limits understanding of how nurses anticipate the ethical, professional, operational, and economic implications of robotic systems before they become part of everyday clinical workflows.

### Conceptual and Ethical Considerations in Nursing Practice

The integration of assistive robots into nursing practice raises conceptual and ethical questions about professional identity, task ownership, accountability, and the boundaries of relational care [4,10,11]. Although these technologies are often framed as supportive tools, their introduction may reshape how nursing work is organized, experienced, and valued.

Existing literature reflects these tensions. Some nurses and care professionals view assistive robots positively, emphasizing their potential to alleviate workload and support care delivery, whereas others express concerns about technical reliability, reduced human interactions, ethical trade-offs, and accountability when care is mediated by machines [10,11,13,16,19]. Practical barriers, including high acquisition and maintenance costs, technical complexity, maintenance requirements, and limited institutional support infrastructure, may further complicate adoption [8,10]. Hesitancy may also reflect anxieties about job displacement or perceived misalignment between robotic functions and core nursing values [6,10,11,17,18].

### Local Context and Study Aim

Nurses play a central role in inpatient care delivery and are directly affected by how assistive robots may reshape workflows, task boundaries, and clinical responsibility. At the same time, the nursing workforce faces sustained pressure from staff turnover, aging populations with complex needs, and increasing care demands [7,8]. Assistive robots and other automated technologies have been proposed as potential tools to support nursing work and care delivery [4,5,10,21], yet successful integration depends heavily on nurses’ engagement, trust, and sense-making [6,13,17-19].

As primary users and workflow mediators, nurses’ involvement in the design, implementation, and evaluation of assistive robots is critical, as they hold firsthand knowledge of clinical demands, patient needs, workflow constraints, and contextual risks [4,5]. Prior research suggests that attitudes toward assistive robots are shaped by familiarity, perceived usefulness, perceived ease of use, and prior or indirect exposure [6,16-20]. However, much of the existing evidence relies on quantitative instruments such as robot attitude or acceptance scales [17-20]. These approaches are useful for measuring acceptance-related constructs but provide limited insight into how nurses interpret, negotiate, and anticipate the implications of assistive robots in everyday inpatient practice.

In the study hospital, assistive robots had been piloted on a limited basis, but were not routinely implemented in inpatient nursing care. Local exposure was primarily limited to demonstrations, institutional briefings, and small-scale pilots involving nonclinical robotic functions, such as logistics support (eg, supply delivery) and task-support activities, such as providing on-demand audio-visual support for patient orientation and educational activities. This created an opportunity to examine how nurses formed anticipatory perspectives in the absence of sustained hands-on experience with assistive robots. This study, therefore, aimed to explore how inpatient nurses conceptualized, interpreted, and anticipated the use of assistive robots in clinical practice, including their perceived benefits, concerns, ethical boundaries, and conditions influencing future adoption.

## Methods

### Study Design

This study used a qualitative descriptive design to explore inpatient nurses’ anticipatory perspectives on assistive robots in clinical care. This design was appropriate because the study sought to generate a practice-near understanding of their expectations, concerns, and perceived implementation conditions in a context where routine exposure to assistive robots was limited. Interview data were analyzed using reflexive thematic analysis.

### Participants and Setting

A convenience sampling strategy was used to recruit registered nurses from 4 general medical inpatient wards in a public tertiary hospital in Singapore. These wards were selected because they were the clinical areas where small-scale pilots, demonstrations, and informal exposure to assistive robots had occurred. At the time of data collection, assistive robots had not been routinely implemented in inpatient nursing care at the study site. Nurses’ exposure was therefore largely indirect and was limited primarily to small-scale pilots and demonstrations involving nonclinical robotic functions, particularly logistics and task-support activities, together with institutional briefings, educational videos, and informal discussions among colleagues. None of these robotic systems were routinely integrated into bedside nursing practice, and participants did not report sustained hands-on experience using assistive robots during their daily clinical work. Consequently, the study explored anticipatory perspectives informed by limited local exposure rather than reflections based on routine clinical use.

Eligible participants were registered nurses working in the selected wards who had direct experience with inpatient nursing workflows. Potential participants were identified through ward rosters and approached by members of the research team during nonclinical hours. Convenience sampling was used because the pool of nurses with contextual exposure to assistive-robot pilots was limited.

The possibility of sampling bias cannot be excluded. Nurses who participated in the interviews could have held stronger preconceptions about assistive robots compared to those who declined. In addition, the sample comprised mainly early-career nurses, which may have shaped the concerns and expectations, and professional identity negotiations reflected in the findings. Nonetheless, the findings may be transferable to similar acute inpatient settings where assistive robots are at an early or anticipatory stage of implementation.

### Researcher Positionality and Reflexivity

The research team comprised registered nurses with at least 5 years of clinical experience in acute inpatient care and prior experience conducting qualitative research. None of the team members had direct hands-on responsibility for implementing assistive robots in clinical practice. This positioned the team as clinically informed about inpatient nursing work but not directly invested in any specific implementation outcome.

Interviews were conducted by 2 members of the research team, TLTC and MWT, both registered nurses who did not hold supervisory, managerial, or appraisal responsibilities for the participants. This helped reduce potential power imbalances during recruitment and interviews.

The researchers recognized that their nursing backgrounds could sensitize them to issues such as workload, patient safety, professional accountability, and relational care, while also potentially shaping how participants’ accounts were interpreted. At the same time, the shared professional background between interviewers and participants was considered likely to facilitate rapport and enable discussion of workplace realities using familiar clinical language. The researchers remained mindful; however, that this insider position could also encourage implicit assumptions about nursing practice or shared understandings that might otherwise have been explored more explicitly during interviews. Interviewers therefore used follow-up and clarifying questions to encourage participants to elaborate on their perspectives rather than assuming shared meanings.

To support reflexivity, the team held regular analytic discussions throughout data collection and analysis. These discussions examined assumptions, considered alternative interpretations, and avoided framing participants’ views as either simple resistance to innovation or uncritical acceptance of technology.

### Procedures

Potential participants were approached in-person by members of the research team during nonclinical hours and provided with an information sheet detailing the study aims and procedures. They were given time to consider their participation. Of the 20 nurses approached, 16 consented to participate, while 4 declined due to scheduling constraints or lack of interest. Written informed consent was obtained from all participants.

A pilot interview was conducted with one registered nurse from a general medical inpatient ward within the same hospital to assess the clarity, flow, and relevance of the interview questions. The pilot also provided the interviewers with an opportunity to familiarize themselves with the interview process and refine their interviewing approach. No substantive revisions to the interview guide were required following the pilot interview, and the same interview guide was subsequently used for all participants. Consistent with qualitative research practice, the pilot interview was not included in the final analysis.

Semistructured, face-to-face interviews were conducted with the 16 participants between October 2023 and June 2024. Interviews took place in a private room within the hospital to ensure confidentiality and minimize interruptions. Both interviewers received the same training to ensure consistency in interview conduct and reduce variation in interview style.

At the start of each interview, participants were provided with a brief description of assistive robots to establish a common frame of reference. Examples included nursing-care assistive robots (eg, medication delivery and patient transport), physically assistive robots (eg, patient lifting and mobility support), and socially assistive robots (eg, companionship and cognitive support). These examples were presented solely to ensure a consistent understanding of the terminology and did not imply that such assistive robots were routinely available at the study hospital. Participants were subsequently invited to describe what they understood by the term “assistive robots” and to reflect on how such technologies might influence nursing practice based on their own observations, indirect exposure, or personal interpretations.

The interview guide explored participants’ perceptions of assistive robots, including perceived benefits, concerns, ethical considerations, and expectations for implementation. Open-ended questions were used, and participants were encouraged to provide concrete examples. Interviews lasted between 60 and 90 minutes. All interviews were audio-recorded with participants’ permission and transcribed verbatim. Identifying information was removed from transcripts to protect participant anonymity.

### Data Adequacy

Recruitment and data collection occurred concurrently with preliminary analysis. Data adequacy was assessed iteratively during data collection, with attention to code saturation and thematic sufficiency rather than to exhaustive meaning saturation. After approximately 14 interviews, no substantially new codes were identified across the main topic areas, and participants’ accounts showed repeated patterns regarding perceived benefits, safety concerns, professional boundaries, and implementation conditions. Two additional interviews were conducted to assess the stability of the developing thematic structure. The final sample of 16 participants was judged sufficient for the study aim, design, and focused clinical context, while recognizing that findings remain contextually situated rather than statistically generalizable.

### Data Analysis

Interview transcripts were analyzed using reflexive thematic analysis as described by Braun and Clarke [22]. The analysis involved iterative phases of familiarization, initial coding, theme development, reviewing, and defining and naming themes.

Consistent with the qualitative descriptive design, the analysis aimed to provide a clinically grounded account of nurses’ perspectives while recognizing that theme development involved active interpretation by the researchers. Themes, therefore, moved beyond simple topic summaries to capture patterns of shared meaning across participants’ accounts, while remaining closely anchored in their descriptions of anticipated experiences with assistive robots.

Initial coding was conducted by 1 author (TCLT) to generate codes that captured patterns of meaning relevant to the study aims. Coding was conducted primarily inductively, while remaining sensitized to concepts identified in prior literature, including perceived usefulness, workflow fit, patient safety, ethical concerns, and professional boundaries. Codes were revised as the analysis progressed and as new interpretations emerged from repeated engagement with the data.

Theme development was interpretive and reflexive rather than based on consensus coding. Provisional themes were generated through repeated engagement with coded data extracts, the full dataset, and emerging analytic interpretations. Regular analytic discussions were held among the research team to interrogate assumptions, explore alternative explanations, and refine the coherence and distinctiveness of themes. These discussions were used to support reflexive interpretation rather than to achieve intercoder agreement.

Themes were finalized based on coherence, distinctiveness, and explanatory relevance to the research questions. An audit trail documenting coding decisions, theme development, and analytic refinements was maintained throughout the process. Data were managed using Microsoft Excel to support systematic coding and documentation.

The study is reported in accordance with the COREQ (Consolidated Criteria for Reporting Qualitative Research) Checklist 1 [23].

### Ethical Considerations

The study was conducted in accordance with the ethical principles outlined in the Declaration of Helsinki. Ethical approval was obtained from the National Healthcare Group Domain Specific Review Board (reference number: 2023/00679). Written informed consent was obtained from all participants prior to the interviews.

Participants were informed that participation was voluntary and that refusal or withdrawal would not result in any adverse consequences, including effects on employment status or performance appraisals. Participants were advised that they had the right to decline to answer any interview questions and to withdraw from the study at any time prior to data analysis.

No reimbursement or incentives were provided for study participation. Participant confidentiality and anonymity were ensured throughout the study. Audio recordings and interview transcripts were stored securely, with access limited to members of the research team. All personally identifiable information was removed during transcription and data analysis. Patients and/or the public were not involved in the design, conduct, reporting, or dissemination plans for this research.

## Results

### Participant Characteristics and Overview of Themes

Demographic characteristics of the 16 participants are presented in Table 1. No participants withdrew after providing informed consent. Participants ranged in age from 21 to 32 years, with a mean age of 26 (SD 2.94) years. The majority of participants were Singaporean citizens or permanent residents (n=11). Five participants were foreign nurses: 4 were from Malaysia and 1 was from Myanmar. The mean duration of professional nursing experience was 34 (SD 2.05) months.

**Table 1.** Characteristics of study participants (N=16).

Characteristic	Value, n (%)
Sex	
Female	16 (100)
Male	0 (0)
Age range (y)	
20‐25	7 (43.8)
26‐30	7 (43.8)
31‐35	2 (12.5)
Designation	
Staff nurse	10 (62.5)
Senior staff nurse	6 (37.5)
Professional nursing experience (y)	
<1	4 (25)
1‐5	11 (68.8)
6‐10	1 (6.3)
Highest nursing education level	
Polytechnic diploma	8 (50)
University degree	8 (50)
Nationality or residency status	
Singapore citizen or permanent resident	11 (68.8)
Foreign nurse: Malaysia	4 (25)
Foreign nurse: Myanmar	1 (6.3)

Four themes were developed from the analysis: (1) anticipatory optimism shaped by indirect exposure; (2) negotiating uncertainty, safety, and professional boundaries; (3) imagining assistive robots as workload and workflow support; and (4) conditions for responsible implementation. Table 2 summarizes the relationship between initial codes, subthemes, and overarching themes. The thematic structure illustrates that participants’ perspectives were not formed through routine use of assistive robots, but through anticipatory sense-making shaped by limited exposure, imagined use, perceived usefulness, safety concerns, professional boundaries, and implementation conditions.

**Table 2.** Development of themes from initial codes to subthemes and overarching themes.

Initial codes or analytic ideas	Subthemes	Overarching themes
Curiosity, demonstrations, media exposure, imagined use, and cautious optimism	Indirect exposure, imagined usefulness, and conditional openness	Anticipatory optimism shaped by indirect exposure
Malfunction, overreliance, patient safety, job displacement, human touch, counseling discomfort, and cost concerns	Safety and reliability, professional role boundaries, relational care limits, and organizational feasibility	Negotiating uncertainty, safety, and professional boundaries
Lifting support, patient turning, transport, basic requests, information retrieval, workload reduction, and staff retention	Physical workload support, routine task support, workflow responsiveness, and organizational benefits	Imagining assistive robots as workload and workflow support
Training, hands-on practice, protected time, phased pilots, technical support, leadership, and champions	Competency development, gradual implementation, support infrastructure, and leadership and buy-in	Conditions for responsible implementation

Table 2 illustrates how participants’ anticipatory perspectives on assistive robots were shaped by perceived usefulness, safety concerns, professional boundaries, workload expectations, and implementation conditions.

### Theme 1: Anticipatory Optimism Shaped by Indirect Exposure

Participants expressed curiosity and cautious optimism toward assistive robots as potential tools to support nursing work and alleviate routine workload. Although most nurses had limited direct exposure to assistive robots in health care, they articulated generally positive expectations about their future role in nursing practice, particularly in relation to physically demanding, repetitive, or time-consuming tasks.

Participants’ perspectives were shaped largely by anticipatory and indirect exposure rather than sustained hands-on use. They drew on demonstrations, informal observations, media portrayals, peer discussions, and imagined future scenarios to consider how assistive robots might function in clinical practice. These indirect forms of exposure contributed to both interest and caution, suggesting that nurses began forming views about assistive robots before routine implementation. One participant stated, “But hopefully in the future I have a robot to help us to turn the patient, take orders from the patient… and engage the patient as well” (P13).

Some participants envisioned assistive robots as intelligent support tools that could enhance efficiency at the point of care by facilitating rapid access to information:

*If the robot… can act like a computer, like Siri… then it can show me immediately. Don’t need to search.*

[P10]

Despite this openness, participants’ optimism was tempered by uncertainty. Nurses emphasized the need for evidence of effectiveness, reliability, and sustainability before fully trusting assistive robots in clinical care. Concerns were raised about the overreliance on technology and the potential consequences of system failure in patient care settings:

*It’s 50–50… it would be really helpful if you can rely on it. But sometimes I’m worried that we might over rely on it.*

[P14]

Others questioned whether assistive robots would genuinely reduce workload or instead introduce additional responsibilities for nurses, particularly if the technology requires ongoing supervision or troubleshooting. One participant stated, “Will it be a helping hand or extra workload for us?” (P13).

Overall, nurses’ accounts reflected openness to innovation alongside a cautious appraisal. Their optimism was not unconditional but grounded in practical concerns about whether assistive robots would be reliable, useful, and compatible with everyday nursing work.

### Theme 2: Negotiating Uncertainty, Safety, and Professional Boundaries

Participants articulated several uncertainties about the integration of assistive robots into nursing practice. A prominent concern was related to job security and role displacement. Some nurses worried that assistive robots might encroach on aspects of nursing work, while simultaneously emphasizing that core nursing functions involving human judgment, accountability, and emotional engagement could not be replicated by machines. One participant stated, “I hope we can use it to relieve the workload, but not totally relying on it… otherwise they would take over our role” (P14).

Concerns about technical reliability were also prominent. Participants highlighted the risk of system malfunction and stressed the importance of maintaining human oversight, particularly in situations where patient safety could be affected. One participant stated, “Assistive robots can malfunction… we as humans should know how to override it and take over” (P07).

Several participants cautioned against excessive reliance on assistive robots, emphasizing that patient safety ultimately remains dependent on nurses’ situational awareness, monitoring, and ability to intervene when needed. One participant stated, “You still need to check the robot because it is about patient safety” (P01).

Participants consistently raised concerns about the potential loss of human touch in nursing care. Emotional support, physical reassurance, and empathetic communication were described as fundamental aspects of nursing that should not be fully delegated to assistive robots. Importantly, nurses did not frame these concerns as a rejection of assistive robots, but rather as boundary-setting around the relational and emotional dimensions of care. One participant stated, “Touch therapy is one aspect that can’t be replaced by assistive robots” (P14).

Some participants expressed discomfort with the idea of assistive robots engaging in emotionally sensitive interactions, such as counseling or responding to patient distress, suggesting limits to the appropriateness of robotic involvement in relational care. One participant stated, “If you have a robot coming to give you counseling, that’s a bit weird… they probably need a human” (P06).

Beyond clinical and ethical considerations, participants framed assistive robots in explicitly economic terms. Bedside nurses-raised concerns about cost-effectiveness, maintenance burden, and return on investment, reflecting a pragmatic awareness of organizational resource constraints and sustainability. One participant stated, “If it’s very expensive and doing very minimal work, then definitely not sustainable” (P06).

Together, these uncertainties show that participants’ concerns were multidimensional. They involved not only whether assistive robots would work technically but also whether they would preserve safety, professional identity, ethical boundaries, organizational feasibility, and relational care.

### Theme 3: Imagining Assistive Robots for Workload and Workflow Support

Despite these uncertainties, participants identified several ways in which assistive robots could positively contribute to nursing practice. Many viewed assistive robots as potential tools for increasing nursing capacity by taking over selected routine or physically demanding tasks. This was perceived as a way to reduce physical strain, conserve cognitive resources, and allow nurses to focus on more complex clinical responsibilities. One participant stated, “It can help reserve our brain power for more critical things” (P08).

Participants anticipated benefits for physically demanding activities, such as patient lifting and transport, which they believed would reduce occupational strain and help prevent injury. One participant stated, “If there is a robot that can help lift a heavy patient, it helps prevent back pain” (P09).

Some participants also linked assistive robots to improved responsiveness to patients’ basic needs. They suggested that assistive robots could help reduce waiting times for simple requests, such as obtaining water or retrieving items, thereby improving the patient experience while allowing nurses to attend to more urgent clinical priorities. One participant stated, “If the robot can help take simple things like water immediately, then the patient will be happy” (P10).

At the organizational level, participants perceived that the use of assistive robots could reap potential operational benefits, including improved productivity, staff satisfaction, and retention. One participant stated, “The staff workload can be a bit lesser… then you can retain your staff” (P02).

Some nurses also noted that visible adoption of assistive robots could enhance the hospital’s public image as innovative and technologically progressive. Importantly, participants consistently framed the benefits of assistive robots as contingent on their integration into nursing practice, emphasizing alignment with nursing workflows, the reduction rather than the addition of workload, preservation of clinical judgment, and the protection of relational care.

### Theme 4: Conditions for Responsible Implementation

Participants emphasized that successful integration of assistive robots would depend on several implementation conditions. Structured and ongoing training was viewed as essential for building confidence and competence, with nurses highlighting the importance of hands-on learning, clear guidance, and opportunities to practice using the technology before being expected to incorporate it into routine care. One participant stated, “If I want to use this robot, I would really want to go for a training session first” (P13).

Protected training time during working hours was considered critical to prevent cognitive overload and ensure meaningful engagement. Participants were concerned that implementation would be less effective if training were added on top of the existing workload or delivered without sufficient opportunity for practice.

Participants also emphasized the importance of phased implementation through small-scale pilots. Gradual introduction was viewed as a way for nurses to assess reliability, understand the robot’s functions, and build trust over time. One participant stated, “Give the staff some time to get used to how reliable the robot is” (P14).

Adequate technical support and rapid troubleshooting were identified as essential to prevent assistive robots from becoming additional burdens rather than supportive tools. One participant stated, “It’s supposed to offload us, not add on” (P08).

Stakeholder buy-in was another key implementation condition. Participants highlighted the role of leadership support, clear communication, feedback mechanisms, and staff champions in encouraging adoption. One participant stated, “You just need a champion… then everybody will hop on to the idea” (P05).

At the same time, nurses acknowledged variability in receptivity to assistive robots, noting differences in comfort with technology among individuals. Participants suggested that tailored training, leadership engagement, and clear expectations may be required to support successful integration. Overall, participants viewed responsible implementation as requiring more than access to the technology itself. It depended on preparation, training, technical infrastructure, leadership support, and governance processes that ensured assistive robots were introduced as supportive tools rather than additional burdens.

## Discussion

### Principal Findings

This study explored inpatient nurses’ anticipatory perspectives on assistive robots in a clinical context where such technologies had been piloted on a limited basis but were not routinely implemented. Participants’ accounts reflected anticipatory acceptance characterized by cautious optimism regarding the potential of assistive robots to support nursing work, alongside concurrent concerns about safety, professional identity, relational care, and implementation feasibility.

The findings extend existing literature by demonstrating that nurses’ views on assistive robots are formed before routine use through anticipatory sense-making. Participants’ perspectives were shaped largely by indirect exposure, including demonstrations, peer discussions, and imagined future use. This preimplementation perspective is important because early interpretations may subsequently influence trust, acceptance, resistance, and implementation trajectories.

Importantly, nurses did not view assistive robots merely as technological devices. Instead, they assessed these technologies in relation to their potential impact on patient safety, professional responsibility, therapeutic relationships, clinical workflows, and the long-term sustainability of health care organizations. Their acceptance of assistive robots was, therefore, contingent upon the extent to which these technologies were perceived as supporting and enhancing, rather than substituting, professional nursing judgment and the relational aspects of care.

### Anticipatory Acceptance Before Routine Implementation

Participants’ cautious optimism is consistent with previous studies showing that nurses and other care professionals often view assistive robots positively when they are perceived to reduce physical workload, support routine tasks, and improve workflow efficiency [4-6,10]. Concerns relating to technical reliability, reduced human interaction, cost, and role displacement align with prior research on nurses’ and care professionals’ attitudes toward assistive robots in health and social care settings [6,13,17-20].

This study adds nuance by focusing on the preimplementation stage. TAMs such as the TAM and the UTAUT emphasize perceived usefulness, perceived ease of use, social influence, facilitating conditions, and intention to use as key determinants of acceptance [14,15]. The Almere model further highlights constructs such as trust, anxiety, perceived sociability, and enjoyment [16]. Building on these concepts, our findings suggest that anticipatory acceptance extends beyond conventional technology assessments of usefulness and usability to include concerns about safety, professional boundaries, relational care, and potential consequences of implementation for everyday work (ie, whether the technology will add to or reduce work).

This finding is consistent with the work by Turja et al [17] which demonstrated that assistive robot acceptance is influenced by factors beyond technical utility. RAM-Care highlights that the intention to use assistive robots is shaped by attitudes, social influence, perceived usefulness, and value alignment, while research on care workers’ readiness for robotization has identified psychological and sociodemographic influences on readiness and acceptance [18]. Our present study extends this literature by showing how nurses reason through these issues before routine implementation, particularly in an acute inpatient environment where assistive robots remain an emerging rather than an embedded technology.

### Task Boundaries, Professional Identity, and Relational Care

Rather than rejecting assistive robots outright, participants appeared to define moral and professional boundaries around their use. Their accounts suggest that acceptance depended less on the technology itself than on whether its intended role aligned with nurses’ expectations regarding appropriate task delegation, clinical judgment, accountability, and relational care. Concerns about “human touch” therefore reflect efforts to preserve aspects of nursing work regarded as fundamentally human, rather than resistance to technology. Viewed in this way, participants’ concerns functioned as a form of boundary-setting through which nurses distinguished tasks that could appropriately be supported by robotic tools from those that should remain primarily human.

This interpretation aligns with the literature suggesting that assistive robots may support selected nursing tasks while also raising questions about the fundamentals of care, professional identity, and relational practice [4-6,10]. Archibald and Barnard [10] argue that assistive robots should be considered in relation to fundamental nursing care goals rather than being treated only as technological innovations. Ethical analyses of assistive robots in health care have also emphasized how robotic systems may alter responsibility, agency, and human interaction in care contexts [11].

Participants’ view that assistive robots should augment rather than replace nursing work is also consistent with local policy directions that position technology as an enabler of care rather than a substitute for professional expertise [24]. Our study findings contribute a frontline perspective by illustrating how nurses define the acceptable limits of robotic assistance in relation to patient safety, accountability, and relational care.

### Safety, Trust, and Human Oversight

Trust emerged as a governance issue rather than merely being an individual attitude. Participants’ accounts suggest that confidence in assistive robots depends on organizational arrangements that ensure reliability, transparency, accountability, and appropriate human oversight. These concerns are consistent with the broader human factors literature on automation bias and complacency, where users may place excessive trust in automated systems or fail to detect errors despite their fallibility [25]. Recent health care–focused work similarly highlights automation bias as a risk associated with the increasing introduction of AI applications into clinical practice when failure modes, accountability, and user oversight are not adequately addressed [26].

For nursing practice, these concerns are especially important because patient safety depends on continuous surveillance, contextual judgment, and timely escalation. Participants not only framed assistive robots as autonomous replacements for nurses but also as tools that should operate under human supervision. This supports a governance approach in which assistive robots are introduced with clear task boundaries, escalation criteria, downtime procedures, incident-reporting mechanisms, maintenance responsibilities, and competency requirements.

The implication is that trust should not be treated as a matter of users’ attitudes alone. Trust must be designed and governed. Nurses are more likely to view assistive robots as acceptable when systems are reliable, transparent in function, supported by technical infrastructure, and embedded within clear accountability arrangements.

### Economic Reasoning and Organizational Readiness

A noteworthy finding was that frontline nurses incorporated organizational and economic considerations into their anticipatory evaluations of assistive robots. Participants questioned whether assistive robots would deliver sufficient benefits to justify investment, whether they could perform meaningful functions within existing workflows, and whether maintenance or troubleshooting would create additional workload. Although economic evaluation and return on investment are often assumed to be managerial concerns, our study findings suggest that frontline nurses also consider organizational sustainability when judging the value of emerging technologies.

This finding extends the literature on robot acceptance by demonstrating that nurses evaluate assistive robots not only through individual acceptance constructs but also through broader considerations such as perceived organizational value. Practical implementation barriers such as cost, technical complexity, and institutional support have similarly been identified in studies of robotic surgery and socially assistive robot implementation [27,28]. Our study’s findings indicate that these considerations are also evident in nurses’ own reasoning even before routine implementation.

The findings also align with implementation science perspectives that view technology adoption as a complex systems process rather than a simple user acceptance issue. The Non-Adoption, Abandonment, Scale-up, Spread and Sustainability (NASSS) framework highlights that adoption and sustainability of health technologies are influenced by the technology, value proposition, adopters, organizational context, wider systems, and adaptation over time [9]. Applying this lens, successful implementation of assistive robots in nursing would depend not only on whether nurses perceive assistive robots as useful but also on whether the organization can provide infrastructure, technical support, workflow redesign, training, maintenance, and credible evidence of value.

### Practical Implications

Our findings suggest that the implementation of assistive robots in inpatient nursing should follow a staged and governance-oriented approach.

First, organizations should conduct workflow mapping before procurement or implementation. This will allow organizations to identify tasks that are repetitive, physically demanding, time-consuming, or logistical, and distinguish them from tasks requiring clinical judgment, emotional support, complex communication, or relational presence. This approach may help ensure that assistive robots are introduced in ways that are most likely to complement, rather than disrupt, nursing work.

Second, implementation should begin with small-scale pilots before wider deployment. Pilot evaluation should assess not only technical performance but also the impact on nursing workload, patient safety, patient experience, staff trust, workflow integration and/or disruption, maintenance burden, and unintended consequences. This is especially important given participants’ concerns that assistive robots could increase rather than reduce workload if poorly integrated.

Third, training should be structured, hands-on, and competency-based. Training should include technical operation, troubleshooting, escalation procedures, downtime management, infection prevention, patient communication, and the limits of robotic assistance. Protected training time may be necessary, as training delivered on top of the routine workload may reinforce perceptions that assistive robots add to the burden rather than reduce it.

Fourth, governance structures should clearly define accountability and support nurses’ involvement throughout the implementation process. Organizations should define who is responsible for monitoring robot performance, responding to malfunctions, reporting incidents, maintaining equipment, reviewing safety data, and deciding when human intervention is required. These arrangements are essential to address participants’ concerns about overreliance, malfunctions, and patient safety.

Fifth, there should be organizational support for nurses’ involvement throughout the implementation stage. Nurses should be engaged early in design, procurement, pilot testing, and evaluation to help identify workflow mismatches, clarify appropriate task boundaries, and address concerns about professional identity and relational care. Nurse champions may support engagement, but champion-led adoption should be accompanied by leadership support, technical infrastructure, and feedback mechanisms.

Overall, implementation strategies should frame assistive robots as supervised tools that augment nursing work, rather than as substitutes for professional judgment or human interaction. Organizations should also establish predefined evaluation metrics to determine whether assistive robots achieve their intended clinical and organizational benefits. These measures may include nursing workload (eg, time spent on routine tasks), operational efficiency (eg, response times and task completion rates), patient safety (eg, robot-related incidents and downtime events), patient experience (eg, patient satisfaction and acceptability), workforce outcomes (eg, nurse satisfaction, perceived workload, and confidence in using assistive robots), and organizational outcomes (eg, robot use, maintenance burden, and service interruptions). Routine monitoring of these indicators would enable organizations to evaluate implementation effectiveness, identify unintended consequences, and refine deployment strategies over time.

### Theoretical Implications

This study contributes to the technology acceptance and robot acceptance literature by foregrounding the anticipatory stage of acceptance. Existing models such as TAM, UTAUT, the Almere Model, and RAM-Care provide useful constructs for understanding perceived usefulness, ease of use, social influence, trust, anxiety, and intention to use [14-17]. The findings suggest that in inpatient nursing, anticipatory acceptance also involves professional and ethical reasoning. Nurses evaluated assistive robots in relation to patient safety, relational care, task legitimacy, accountability, workload implications, and organizational sustainability.

The study also contributes to the sociotechnical understanding of health care technology adoption. Participants evaluated assistive robots within broader clinical, organizational, and professional contexts rather than as isolated technological artifacts. Acceptance was shaped by anticipated interactions between technology and existing staffing pressures, workflow demands, patient needs, professional values, maintenance systems, and leadership support. Taken together, our findings support the view that the implementation of assistive robots represents a sociotechnical change in nursing work rather than simply the introduction of a new device.

### Limitations

This study has several limitations. First, participants were recruited from 4 general medical inpatient wards in one public tertiary hospital in Singapore where assistive robots had been piloted on a limited basis but were not routinely embedded in practice. The findings therefore reflect anticipatory perspectives rather than experiences of sustained use. Nurses’ views may change after direct and repeated interaction with assistive robots in routine care.

Second, convenience sampling may have introduced selection bias. Nurses with stronger views about assistive robots may have been more willing to participate than those who declined participation.

Third, the sample consisted mainly of early-career nurses, which may have shaped the emphasis on professional identity, role security, and confidence in managing emerging technologies. More experienced nurses, advanced practice nurses, and nurse leaders may place greater emphasis on clinical governance, service redesign, workflow integration, and organizational implementation considerations. Future studies should therefore include participants across different career stages and nursing roles.

Fourth, the study was conducted in a digitally enabled hospital context, which may limit transferability to settings with different staffing models, technological maturity, infrastructure, or organizational readiness. Although the findings may be relevant to similar acute inpatient settings where assistive robots are at an early stage of implementation, they should not be assumed to represent all nursing contexts.

Future research should examine how nurses’ perceptions evolve after sustained exposure to assistive robots and should incorporate the perspectives of multiple stakeholders. Given participants’ concerns about human touch, counseling, and patient experience, future studies should also explore how patients and family caregivers perceive acceptable uses for assistive robots in inpatient care, particularly for activities involving communication, emotional support, and relational aspects of care. Longitudinal and comparative studies are also needed to evaluate the effects of assistive robots on workload, patient safety, patient experience, staff trust, cost-effectiveness, and long-term sustainability.

### Conclusions

This study demonstrates that inpatient nurses form nuanced anticipatory judgments about assistive robots before routine implementation. Participants expressed cautious optimism about the potential for assistive robots to reduce workload and support selected nursing tasks, but their acceptance was conditional on safety, reliability, workflow fit, human oversight, relational care, cost-effectiveness, and organizational readiness.

The findings suggest that assistive robot implementation should not be viewed as a purely technical undertaking. Responsible and successful integration will depend on early nurse engagement, workflow-sensitive design, phased piloting, structured training, clear governance, and safeguards that ensure assistive robots augment rather than undermine nursing practice. Nurses’ acceptance depends on whether robotic systems are perceived as compatible with professional judgment, patient-centered care, ethical accountability, and practical realities of inpatient work. By foregrounding the anticipatory stage of acceptance, this study highlights how nurses evaluate assistive robots not merely as technical tools, but as potential change agents that may shape professional work, relational care, safety, accountability, and organizational resource use.

## Supplementary material

10.2196/91106 Checklist 1 COREQ Checklist

## Abbreviations

COREQ: Consolidated Criteria for Reporting Qualitative Research
NASSS: Non-Adoption, Abandonment, Scale-up, Spread and Sustainability
RAM-Care: Robot Acceptance Model for Care
TAM: Technology Acceptance Model
UTAUT: Unified Theory of Acceptance and Use of Technology

## References

[1] World Robotics 2025 report – SERVICE ROBOTS – released by IFR. International Federation of Robotics (IFR).

[2] Alboul L, Dimitrova M, Lekova A, Kaburlasos VG, Mitrouchev P (2023). Editorial: emerging technologies for assistive robotics: current challenges and perspectives. Front Robot AI.

[3] Javaid M, Haleem A, Pratap Singh R, Rab S, Suman R, Kumar L (2025). Utilization of robotics for healthcare: a scoping review. J Ind Integr Mgmt.

[4] Christoforou EG, Avgousti S, Ramdani N, Novales C, Panayides AS (2020). The upcoming role for nursing and assistive robotics: opportunities and challenges ahead. Front Digit Health.

[5] Kangasniemi M, Karki S, Colley N, Voutilainen A (2019). The use of robots and other automated devices in nurses’ work: an integrative review. Int J Nurs Pract.

[6] Papadopoulos I, Koulouglioti C, Ali S (2018). Views of nurses and other health and social care workers on the use of assistive humanoid and animal-like robots in health and social care: a scoping review. Contemp Nurse.

[7] Poon YSR, Lin YP, Griffiths P, Yong KK, Seah B, Liaw SY (2022). A global overview of healthcare workers’ turnover intention amid COVID-19 pandemic: a systematic review with future directions. Hum Resour Health.

[8] Mitchell E, Walker R (2020). Global ageing: successes, challenges and opportunities. Br J Hosp Med (Lond).

[9] Greenhalgh T, Wherton J, Papoutsi C (2017). Beyond adoption: a new framework for theorizing and evaluating nonadoption, abandonment, and challenges to the scale-up, spread, and sustainability of health and care technologies. J Med Internet Res.

[10] Archibald MM, Barnard A (2018). Futurism in nursing: technology, robotics and the fundamentals of care. J Clin Nurs.

[11] Stahl BC, Coeckelbergh M (2016). Ethics of healthcare robotics: towards responsible research and innovation. Rob Auton Syst.

[12] Mucchiani C, Cacchione P, Johnson M, Mead R, Yim M (2021). Deployment of a socially assistive robot for assessment of COVID-19 symptoms and exposure at an elder care setting. Proc IEEE Int Conf Robot Hum Interact Commun (RO-MAN).

[13] Kang HS, Koh IS, Makimoto K, Yamakawa M (2023). Nurses’ perception towards care robots and their work experience with socially assistive technology during COVID-19: a qualitative study. Geriatr Nurs.

[14] Davis FD (1989). Perceived usefulness, perceived ease of use, and user acceptance of information technology. MIS Q.

[15] Venkatesh V, Morris MG, Davis GB, Davis FD (2003). User acceptance of information technology: toward a unified view. MIS Q.

[16] Heerink M, Kröse B, Evers V, Wielinga B (2010). Assessing acceptance of assistive social agent technology by older adults: the Almere model. Int J Soc Robotics.

[17] Turja T, Aaltonen I, Taipale S, Oksanen A (2020). Robot acceptance model for care (RAM-care): a principled approach to the intention to use care robots. Inf Manage.

[18] Turja T, Taipale S, Kaakinen M, Oksanen A (2020). Care workers’ readiness for robotization: identifying psychological and socio-demographic determinants. Int J Soc Robotics.

[19] Andtfolk M, Nyholm L, Eide H, Rauhala A, Fagerström L (2022). Attitudes toward the use of humanoid robots in healthcare—a cross-sectional study. AI Soc.

[20] Felding SA, Koh WQ, Teupen S, Budak KB, Laporte Uribe F, Roes M (2023). A scoping review using the Almere model to understand factors facilitating and hindering the acceptance of social robots in nursing homes. Int J Soc Robotics.

[21] National Academies of Sciences, Engineering, and Medicine (2021). The Future of Nursing 2020-2030: Charting a Path to Achieve Health Equity.

[22] Braun V, Clarke V (2006). Using thematic analysis in psychology. Qual Res Psychol.

[23] Tong A, Sainsbury P, Craig J (2007). Consolidated criteria for reporting qualitative research (COREQ): a 32-item checklist for interviews and focus groups. Int J Qual Health Care.

[24] Keynote Address by Mr Ong Ye Kung, Minister for Health, at the Singapore Health &amp; Biomedical Congress 2024, on Thursday 10 October 2024, Singapore Expo. Ministry of Health.

[25] Wickens CD, Clegg BA, Vieane AZ, Sebok AL (2015). Complacency and automation bias in the use of imperfect automation. Hum Factors.

[26] Abdelwanis M, Alarafati HK, Tammam MMS, Simsekler MCE (2024). Exploring the risks of automation bias in healthcare artificial intelligence applications: a Bowtie analysis. J Saf Sci Resilience.

[27] Lawrie L, Gillies K, Duncan E, Davies L, Beard D, Campbell MK (2022). Barriers and enablers to the effective implementation of robotic assisted surgery. PLoS One.

[28] Zafrani O, Nimrod G, Krakovski M, Kumar S, Bar-Haim S, Edan Y (2024). Assimilation of socially assistive robots by older adults: an interplay of uses, constraints and outcomes. Front Robot AI.

